# Sub-chronic Dermal Toxicity of Silver Nanoparticles in Guinea Pig: Special Emphasis to Heart, Bone and Kidney Toxicities 

**Published:** 2013

**Authors:** Mitra Korani, Seyed Mahdi Rezayat, Sepideh Arbabi Bidgoli

**Affiliations:** a *Deparment of Pharmacology, Faculty of Medicine, Tehran University of Medical Sciences, Tehran, Iran.*; b *Depatment of Nanotechnology, Faculty of Advanced Sciences and Technology in Medicine, Tehran University of Medical Sciences, Tehran, Iran,*; c *Department of Toxicology and Pharmacology, Pharmaceutical Sciences Branch, Islamic Azad University, Tehran, Iran. *

**Keywords:** Ag NPs, Nanosilver, Cardiotoxicity, Osteotoxicity, Nephrotoxicity, sub-chronic dermal toxicity, Silver nanoparticles

## Abstract

Silver nanoparticles (Ag NPs) have been widely used as new potent antimicrobial agents in cosmetic and hygienic products. Present study compares the tissue levels of Ag NPs in different organs of Guiana Pigs quantitatively after dermal application and analysis the morphological changes and pathological abnormalities on the basis of the Ag NPs tissue levels.

Before toxicological assessments,the size of colloidal nanosilver was recorded by X-Ray Diffraction and Transmission Electron Microscope tests and the sizes of samples were recorded in sizes less than 100 nm. For toxicological evaluation, male guinea pigs were exposed to three concentrations of Ag NPs (100, 1000 and 10000 ppm) according to acute pretests for further assessments in subchronic model in a period of 13 weeks .

A close correlation between dermal exposure and tissue levels of Ag NPs was found (p < 0.05) and tissue uptakes happened in dose dependent manner with the following ranking: ki dney>muscle>bone>skin>liver>heart >spleen. In histopathological studies, severe proximal convoluted tubule degeneration and distal convoluted tubule were seen in the kidneys of the middle and high-dose animals. Separated lines and marrow space narrow were determined as two major signs of bone toxicities which observed in three different dose levels of Ag NPs. Increased dermal dose of Ag NPs caused cardiocyte deformity, congestion and inflammation.

The three different Ag NPs concentration gave comparable results for several endpoints measured in heart, bone and kidney, but differed in tissue concentrations and the extent of histopathological changes. It seems that Ag ions could be detected in different organs after dermal exposure ,which has the potential to provide target organ toxicities in a time and dose dependent manner.

## Introduction

Nowadays engineered nonmaterial has attracted a great deal of attention due to its important changes in basic properties of materials which have given birth to vast technological and economical growth in a number of industrial sectors worldwide. In fact ,nano-materials are expected to become the cornerstone of many industrial sectors especially in healthcare and cosmetic products in the near future (1). Silver nanoparticles (Ag NPs) are a group of them which have become one of the most widely used nonmaterial in consumer products because of its antimicrobial and antiseptic properties, but public concerns over its potential adverse effects (2) has encouraged scientists to focus on their safety profiles too . 

For nanotechnology based consumer products, a wide variety of globally available products have been introduced to the market and the identified nanotech goods have a rise of 279% when compared to the first inventory in 2006 (3). This rise clearly indicates that how fast the market of nano containing health products is growing. According to the recent market surveys more than 25% of this fast growing market belongs to Ag NPs (4) and the largest number of Ag NPs containing consumer products are in the categories of textiles and shoes (5), personal care ,cosmetics, clothing and skin care as subcategories(4) which expose to the human by dermal application. Moreover silver nanoparticles have recently been used for diverse medical applications such as surgical sutures and silver-coated medical devices (6). 

Though nanosilver-based health products have received approval for clinical application and good control of infection has been achieved, their dermal toxicity is still a topic of concern. The potential cytotoxicity of Ag NPs in human epidermal keratinocytes and their inflammatory and penetrating potential into porcine skin *in-vivo *were assessed recently (7) but the systemic toxicity of Ag NPs via dermal application had remained undetermined before our report in 2011 (8). We showed that Ag NPs could be found in skin, liver, and spleen of guinea pig after dermal application and may result in slight damages in liver, spleen, and skin. Due to the importance of dermal exposure to Ag NPs in different health products and due to the lack of knowledge about the possible toxic effects of nanosilver in other organs including heart, kidney and bone, we have decided to follow the histopathological effects of Ag NPs on these organs after dermal application and compare the possible toxicity of Ag NPs in Guiana pig by subchronic test. 

## Experimental

*Silver nanoparticles (Ag NPs)*

As previously described (8), Ag NPs were purchased from Quantum sphere company, USA. Three different aqueous solutions (100, 1000 and 10000 ppm) were provided kindly by Dr. K Gilani in the Pharmaceutics Lab., Faculty of Pharmacy, Tehran University of Medical Sciences.

*Experimental animals and housing conditions*

In present study, 60 experimental male Hartley-albino guinea pigs were obtained from Pasteur Institute of Iran .Their ages were between 5 to 6 weeks and their body weights were between 350-450 g. Each three guina pigs were housed in stainless steel cages and allowed to adapt to the conditions of the animal house for 14 days before the experiments. The animals were maintained on a 12 h dark/light cycle at about 22 ± 3 ^o^ C and allowed free access to standard laboratory diet, vitamin C and tap water and libitum during the experiments. Hair of animals were shaved in the area where silver nitrate and Ag NPs were used. They were randomly divided to five treatment groups, each one contained 12 animals. One to AgNo_3_ , three to Ag NPs and one to control group. All animal studies were conducted according to the US National Institute of Health (NIH publication no. 85-23, revised 1985) guidelines (9). The studies were conducted in accordance with the laws and regulations of governing authorities in Iran . The study design was approved by Tehran University of Medical Science**s, **Animal Care and Use ethical Committee 

*TEM and XRD tests*

To detect the extent and size of Ag NPs, transmission electron microscopy (TEM) and X-ray diffraction (XRD) were used by a standard equipment( Siemens with Cu source, 40 K V and 30 mA ).Sample patterns were determined at 5°- 75° (2θ )(10).

*Subchronic dermal toxicity studies*

Subchronic dermal toxicity test was performed in compliance with the OECD guideline No. 411.After performing the acute test and estimating the necessary doses for subchronic toxicity assessments, the test substance at low dose group (100 ppm), middle dose group (1000 ppm) and high dose group (10000 ppm) of nanosilver and a 100 μg/mL of the solution of AgNo_3_ were applied to 10% of the body surface area of experimental animals. The other untreated portions of treated animals were kept as a negative control. The observation period of this part was 13 weeks. In the meanwhile clinical signs were observed and weights were recorded 2 times /week. The recording items were divided to three categories: Cage side observations, neurological and physical examination. 

*Determination of tissue levels of Ag NPs*

Tissues were digested with nitric acid by using flameless method. Then the tissue concentrations of silver were analyzed by using an atomic absorption spectrophotometer equipped with a graphite furnace (Perkin Elmer 5100ZL, Zeeman Furnace Module, USA) based on the Abraham TW *et al.*’method (11). 

*Pathological studies*

Heart ,kidney and bone were three major organs which were focused in this study .These organs were removed from 3 animals/ group for histopathological studies at three intervals: baseline, midpoint and endpoint . The tissues were fixed in 10% buffered formalin and dehydrated in graded series of alcohol, cleared in xylene and embedded in paraffin wax. Multiple sections from each block were prepared at 5 μm and stained with haematoxylin and eosin (H&E) for histopathological studies. 

## Results

*TEM studies*

**Figure 1:**
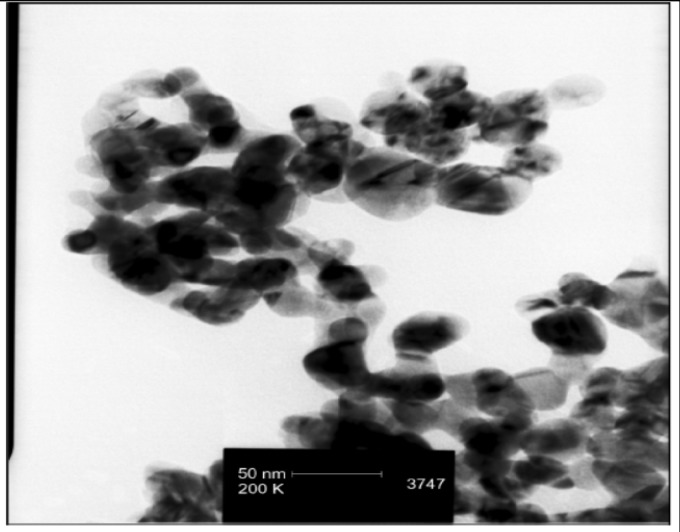
TEM image of silver nanoparticles show that the particles are < 100 nm

By using TEM analysis, nanosilver particles were detected in sizes less than 100 nm (Figure 1). 

*XRD examination*

**Figure 2:**
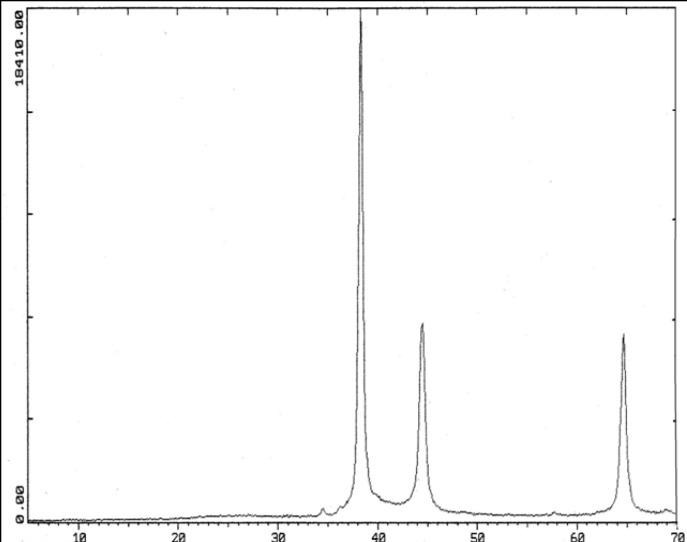
In XRD test, peaks at 38, 44 and -64.5 degrees indicates existence of nanosilver

Picks of Ag NPs by XRD were observed in the ranges of 30, 44, 64/5 degrees (^2θ^) (Figure 2). 

*Mortality rate*

No mortality was recorded during dermal application of different concentrations of Ag NPs in doses up to 10000 μg/mL in preliminary study. Therefore we considered it as a practically non toxic agent in the acute dermal exposure . 

*TEM and XRD*

Ag NPs were observed in sizes of less than 100nm by TEM .Their picks were occurred at 2θ values of 38, 44,64/5 degrees in the XRD . TEM and XRD characteristics of Ag NPs were presented in our previous study (8). 

*Food and water consumption and weight changes*

Food and water consumption was not significantly different among control and treatment groups (p=0.085*, *p*=*o.087 respectively) .Weights of animals from all three different treated groups were recorded two times weekly. No significant weight changes were detected during the study. 

*Survival and clinical signs*

All animals were survived during this study in both cases and control groups. Out of different daily cage side observations no clinical sign of toxicity was recorded in dose groups and control. 

*Tissue levels of Ag NPs*

**Figure 3:**
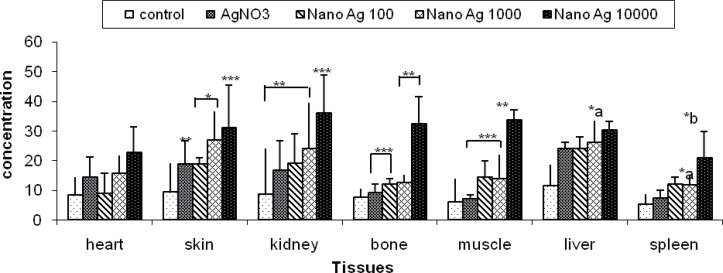
Differential levels of AgNo_3_ and Ag NPs in different tissues according to dermal application in Guinea pig.* p < 0.05,** p < 0.01,*** p < 0.001. a: Significant difference vs. AgNO_3_. b: Significant difference vs. nanosilver1000 μg/mL

A close correlation between dermal exposure and tissue levels of Ag NPs was found in this study (p < 0.05) (Figure 3). After comparing the tissue levels of Ag NPs in different target the skin and kidneys in high dose treated animals. The tissue uptake of Ag NPs was happened in dose dependent manner. Although the heart uptake of Ag NPs via dermal application was not significantly higher even in the high dose group (13.68+8.46 vs.), when it compared with normal group, other organs showed significantly higher levels in the following manner: kidney>muscle> bone>skin.>Liver>heart >spleen (Table 1). 

**Table 1:** Tissue levels of Ag NPs in comparison to AgNo_3_ and negative control (ng/g)*.*

**Dose Groups **	**Heart ** **(n=26) **	**Skin ** **(n=22) **	**Kidney ** **(n=28) **	**Bone ** **(n=27) **	**Muscle ** **(n=28) **	**Liver ** **(n=27) **	**Spleen ** **(n=26) **
Control negative	8.46+ 6.85	9.36 +2.28	8.78 +2.64	7.7736 +1.96	6.109+1.06	11.5+1.29	5.36+1.37
Control Positive (AgNO3)	14.34 + 6.85	18.84+ 7.72	16.6 +9.92	9.3377+2.75	7.124+1.39	24.06+2.05	7.50+2.46
Ag NPs 100	9.09 +6.85	18.84+2.002	19.03+9.92	12.025+1.97	14.316+5.68	24.06+3.80	12.07+2.46
Ag NPs 1000	15.65+5.96	29.96+9.69	24.06+15.36	12.557+2.75	14.026+7.84	26.148+7.21	11.88+3.23
Ag NPs 10000	22.66+8.73	31.02+14.3	35.95+12.94	32.325+9.1	33.63+3.31	30.324+2.84	20.87+8.85

*Toxic responses of bone*

**Figure 4:**
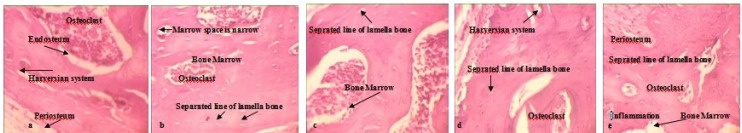
Comparison of the osteotoxic effects between AgNo_3_ and Ag NPs in treated animals detected by H&E staining (10x). a: Negative control shows normal bone marrow, the osteon or Haversian system and Periosteum b: AgNO_3_ treated bone, c: Low dose Ag NPs treated bone,d: Medium dose Ag NPs treated bone, e: High dose Ag NPs treated bone

Bone samples of 27 animals were observed and scored according to the classifications in the Table 2 .Abnormal inflammatory responses were found in all treated groups (Figure 4a-e) and osteoclasts were formed in these animals in a dose dependent manner. Separated Lines and marrow Space narrow were observed in three different dose levels of Ag NPs when the alterations were compared with negative group. (Figure 4b-e). More details about Ag NPs induced bone toxicities are listed in Table 2. 

**Table 2:** Ag NPs induced bone toxicity after dermal application in Guina pig

**Dose Groups**	**Inflammation **	**Osteoclasts**	**Separated Lines**	**Marrow Space narrow**
Control negative	-	-	-	-
Control Positive (AgNO_3_)	+	+	+	+
Ag NPs (100	+	+	+	+
Ag NPs 1000	+	++	+	+
Ag NPs 10000	+	+++	++	++

Severe (+++), moderate (++), mild (+), none(-)

*Toxic responses of heart*

**Figure 5:**
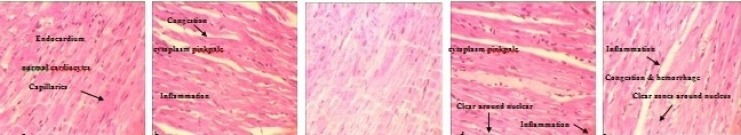
Comparison of the cardiotoxic effects between AgNo3 and Ag NPs in treated animals detected by H&E staining (10x). a: Negative control shows endocardium, normal cardiocytes and capillaries. b: AgNO_3_ treated heart shows congestion and hemorrhage, inflammatory tissues and cytoplasm pink pale, c: Low dose Ag NPs treated heart showed no abnormality, d: Medium dose Ag NPs treated heart shows cytoplasm pink pale and Clear zones around nucleus, e: High dose Ag NPs treated heart shows inflammation, congestion, hemorrhage and cytoplasm pink pale and Clear zones around nucleus

After evaluating the tissue samples of 26 animals, toxic responses were categorized on the basis of the following abnormalities: inflammation, presence of clear zone around nucleus, cardiocyte deformities, congestion and hemorrhage. As we showed in Table 3, abnormal changes were detected in dose groups as well as AgNO_3_ group. However all 4 major signs of toxicity were magnified in the high dose group. Increased dermal dose of Ag NPs caused cardiocyte deformity (Figure 5c-d). More details about histopathological changes in the heart of animals are listed in Table 3.

**Table 3:** Ag NPs induced heart toxicity after dermal application in Guinea pig

**Congestion & hemorrhage**	**Clear zones around nucleus**	**Cardiocyte deformity **	**Inflammation**	**Dose Groups**
-	-	-	-	Control negative
+	-	+	+	Control Positive (AgNO3)
+	+	+	+	Ag NPs (100
+	+	++	+	Ag NPs 1000
++	++	++	++	Ag NPs 10000

Severe (+++), moderate (++), mild (+), none(-)

*Kidney*

**Figure 6:**
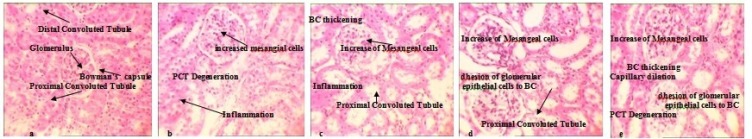
Comparison of the nephrotoxic effects between AgNo_3_ and Ag NPs in treated animals detected by H&E staining (10x). a: Negative control shows BC (Bowman's capsule) DCT ( Distal Convoluted Tubule), PCT ( Proximal convoluted tubule) and GL (Glomerulus) b: AgNO_3 _treated kidney shows Capillary dilation, PCT degeneration and inflammatory responses, c: Low dose Ag NPs treated kidney shows Inflammation, Adhesion of glomerular epithelial cells to BC, BC thickening, PCT Degeneration and increased Mesangial cells, d: Medium dose Ag NPs treated kidney shows Inflammation, Adhesion of glomerular epithelial cells to BC, BC thickening, PCT Degeneration and increased Mesangial cells e: High dose Ag NPs treated kidney shows the same but more severe toxic effects

After exact evaluation of 28 kidneys from treated animals and comparing the levels with control group, six major toxic responses were observed and scoring was performed according to the following classification: Inflammation, gluomeral adhesion to Bowman’s capsule, Proximal convoluted tubule Degeneration, Capsular thickening, membranous thickening and increased mesangial cells (Figure 6). As we showed in Table 4, inflammatory reactions and glomeral adhesion to Bowman›s capsule were identified in all dose groups. These reactions were magnified in a dose-dependent manner (Figure 6c-e). Except these toxic reactions, increased mesangial cells, increased membranous thickening and increased capsular thickening were detected too (Figure 3b-e). The highest levels of degeneration proximal convoluted tubule and distal convoluted tubule were seen in the middle and high-dose groups. 

**Table 4:** Ag NPs induced nephrotoxicity after dermal application in Guinea pig

**Dose Groups**	**Inflammation **	**PCT Degeneration**	**Adhesion of glomerular epithelial cells to BC**	**Capsular thickening**	**Membranous ** **thickening **	**increased Mesangial cells **
Control negative	-	-	-	-	-	-
Control Positive (AgNO3)	+	++	-	+	-	+
Ag NPs 100	+	++	+	+	-	+
Ag NPs 1000	+	++	+	+	+	+
Ag NPs 10000	+	+++	++	++	++	++

BC: Bowman's capsule, DCT : Distal Convoluted Tubule, PCT: Proximal convoluted tubule,GL: Glomerulus

## Discussion

Most of the available information on the toxicity mechanisms of Ag NPs comes from *in-vitro *studies (12, 13), with only limited data from *in-vivo *studies (14) .Therefore it is necessary to focus on the organ toxicity of Ag NPs in animal models which was the major concern of present study. Existing information has proposed three main mechanisms for the toxicity of Ag NPs: oxidative stress (15), DNA damage (16) and cytokine induction (14). It is demonstrated also that Ag NPs could be internalized by scavenger receptors, trafficked to cytoplasm and induce toxicity by releasing Ag ions .Although Ag NPs cause toxic responses according to the above mechanisms ,the presence of Ag ion-reactive, thiol-containing compounds are predisposing factors in AgNP toxicity (17). We tried in the present study to determine the tissue levels of Ag ions in AgNo_3_, Ag NPs and control groups by Atomic absorption spectroscopy because quantitative image analysis demonstrated that intracellular dissolution of Ag NPs occurs about 50 times faster than in water (17) and Ag has systemic absorption via dermal application . 

After comparing tissue levels of Ag NPs which was closely associated with administered doses of AgNPs and AgNO_3_ in different target organs, the highest tissue uptake was found in the kidney. One recent study showed that when mice were treated with AgNPs 1 mg/kg for 14 days by oral administration, small-sized AgNPs were distributed to organs including brain, lung, liver, kidney and testis ,while large-sized Ag (323 nm) were not detected in those tissues (18). We have shown in this study how the small size Ag NPs and large size AgNO_3_ distributed to different organs including kidney, liver, muscle, spleen , bone, skin, and heart, which detected by Atomic absorption spectroscopy for determining the tissue levels of Ag NPs s. We showed recently that how AgNPs affect liver, skin and dermis (8) and muscle (19) by dermal administration.Here we have shown the tissue abnormalities of heart, kidney and bone on the basis of dose administration, tissue concentrations and histopathological examinations. 

Most of toxicological studies on Ag NPs are limited to inhalational (20) or oral administration (21), whereas the dermal exposure is the major rout of exposure in human population (2). In last studies animals which were exposed to AgNO_3_, showed minimal pulmonary inflammation or cytotoxic reaction following sub-acute exposures .But longer term exposures with higher body burdens of Ag NPs via dermal exposure are needed to ensure that there are no chronic effects and also to evaluate possible translocation of Ag NPs to other organs. 

In the second part of this study, we focused on the histopathological effects of different concentrations of Ag NPs by dermal application on the bone, heart and kidney in the comparison to AgNO_3_ for the first time. Present study clearly showed that dermal contact to Ag NPs may cause histopatholgical abnormalities in the kidney, bone and heart of animals which could be magnified by increased concentration in longer term exposures. More data from few *in-vivo *studies on the toxicity of Ag NPs supports this fact that exposure to Ag NPs can result in effects in different major organs (21). Thus it is very important to describe its descriptive toxic effects on each organ before determining the exact mechanism of toxic effects. All *in-vivo *studies which were summarized the toxic effects of Ag NPs used different formulations of AgNPs. They were mostly generated in the laboratory and some were purchased commercially but very few studies have evaluated the systemic toxic effects of AgNPs ‘leached’ from current commercial products in a dermal exposure model. The present study has focused on the systemic toxicity of AgNPs via dermal administration, determined the tissue levels of AgNPs at three conventional doses after dermal application and examined the histopathological effects of Ag NPs on the heart, bone and kidneys of treated Guinea pigs in a subchronic model. 

Toxicity of nanoparticles including Ag NPs depends on many factors including size, shape, chemical composition, surface area, surface charge (18). Rout of administration and repeated dose exposure are two other important factors which were considered in this study. Each significant size change of the silver particles may change its interaction profiles with subcellular components. In fact the higher levels of in vitro hemolysis were observed with silver nanoparticles when compared with micron-sized particles because of their greater surface area, increased silver ion release, and direct interaction with RBCs (22). 

By the administration of Ag NPs (100 ppm, 1000 ppm and 10000 ppm), adverse impacts on kidney ,heart and bone were observed especially in medium and high dose Ag NPs treated groups when compared with micron sized Ag by histopathological analysis. Some reports have proved that many medical devices loaded with silver could release silver ions (Ag^+^) which could translocate in blood circulation and accumulate in some organs such as liver and kidney. It may induce hepatotoxicity or renal toxicity and may lead to death in some situation extremely exposed to a certain dose of Ag (13). We showed already the hepatotoxic effects of Ag NPs in our recent study (8) and we proved in present study that Ag NPs with close properties to silver ions could translocate in the body via dermal application and accumulate in the kidney up to 35.95+12.94 ng/g, (Table 1). Although the administered concentrations of Ag NPs were high and unrealistic, but as far as we know this is the first study on the determination of tissue levels of Ag NPs after dermal exposure. It seems necessary to conduct the same study on lower doses and find the NOAEL (No observable adverse effect level ) of Ag NPs on the basis of histopathological damages.

## Conclusions

Except target organs of Ag NPs which were showed in our previous studies (8), (19) ,the toxicity potentials of Ag NPs on three new target organs including kidney, heart and bone were determined in this study. Although a NOAEL of 30 mg/kg and LOAEL of 125 mg/kg (23) were suggested for oral administration of AgNPs in rats, doses of o.1 mg/kg (100 mcg) were not safe dose for dermal application. Although no sign of mortality was detectable in all treatment groups, significant dose-dependent histopathological changes were found in all treatment groups comparing to controls .Present results indicate that exposure to more than 0.1 mg/kg of Ag NPs may result in slight kidney, heart and bone damage. 

Considering to the present findings, it is necessary to find the association between the period of exposure and histopathological changes with lower doses in different time periods. It is also highly recommended to detect the role of shape and particle size on the toxicity profile of AgNPs by different routs of administration in the future studies.
